# Antipsychotics and mood stabilizers on risk for hepatic failure in people with schizophrenia and bipolar disorder

**DOI:** 10.1192/j.eurpsy.2022.1864

**Published:** 2022-09-01

**Authors:** M.F. Tascón Guerra, M. Pérez Fominaya, M.V. López Rodrigo, A. Osca Oliver, M. Palomo Monge, A. Costoso López, V. Ros Font

**Affiliations:** 1 Hospital Nuestra Señora del Prado, Psiquiatria, Talavera de la Reina, Spain; 2 Hospital Nuestra Señora del Prado, Psiquiatría, Talavera de la Reina, Spain

**Keywords:** neuroleptic side effects, clozapine, psychopharmacology, hepatitis

## Abstract

**Introduction:**

Patients with severe mental disease have a considerably shorter lifespan than the general population. The majority of psychiatric drugs are metabolized by the liver. Cytochromes play a central role, interactions between drugs are expected. Neuroleptics are frequently associated with weight gain, steatosis development, elevation of liver enzymes and rare acute cytolytic hepatitis, particularly with clozapine and olanzapine. Mood stabilizers, like Valproate classically gives mitochondrial steatosis with potentially important damages, and also possible acute liver failure.

**Objectives:**

This case presents a 56-year-old patient, previously diagnosed of schizoaffective disorder, with chronic psychotic symptoms that showed high drug resistance. She had been treated in the past with most common antipsychotic drugs with no clinical response. While being in treatment with valproate and olanzapine, she was started on clozapine while olanzapine removed. Two weeks later she developed Acute Pharmacologic Hepatitis with mild liver failure.

**Methods:**

Physical examination was normal. Mental exam revealed presence of delusion. Blood tests showed: hyperbilirubinemia and mil coagulopathy. Clozapine dose was reduced and valproate was suspended.

**Results:**

The patient showed a substantial improvement of hepatic damage. Delusions are active after 12 weeks of treatment with clozapine.

**Conclusions:**

Psychiatric disorders and liver illnesses are entangled in multiple ways. Screening for liver diseases is essential in order to prevent liver complications in patients receiving psychotropic medications. Further investigation of combinations of agents such as mood stabilizers and atypical antipsychotics may yield valuable insights into the potential of combination therapies to enhance clinical outcomes in patients with Severe Mental Disease.

**Disclosure:**

No significant relationships.

